# Aqueous Extract of *Lycium ruthenicum* Murray Attenuates Neuroinflammation in C57BL/6J Mice Induced by High-Fat and High-Fructose Diet Through Regulating Gut Microbiota and Bile Acid Metabolism

**DOI:** 10.3390/foods13233812

**Published:** 2024-11-26

**Authors:** Xia Fan, Wei Dong, Yujie Huang, Yifan Shu, Yamei Yan, Jia Mi, Lu Lu, Xiaoxiong Zeng, Youlong Cao

**Affiliations:** 1 College of Food Science and Technology, Nanjing Agricultural University, Nanjing 210095, China; fanxia@njau.edu.cn (X.F.); 2021208003@stu.njau.edu.cn (W.D.); 2019208020@njau.edu.cn (Y.H.); 2020108061@stu.njau.edu.cn (Y.S.); 2 Institute of Wolfberry Engineering Technology, Ningxia Academy of Agriculture and Forestry Sciences, Yinchuan 750002, China; yanyamei@163.com (Y.Y.); 2023208003@stu.njau.edu.cn (J.M.); 2023208002@stu.njau.edu.cn (L.L.); 3 National Wolfberry Engineering Research Center, Yinchuan 750002, China

**Keywords:** *Lycium ruthenicum* Murray, neuroinflammation, gut microbiota, bile acids

## Abstract

The aqueous extract of *Lycium ruthenicum* Murray (LRE) could attenuate neuroinflammation in mice induced by a high-fat and high-fructose diet (HFFD). Moreover, LRE could adjust bile acid (BA) metabolism and the gut microbiota. Behavioral tests revealed that LRE prevented HFFD-induced cognitive deficits. The treatment of LRE resulted in a decreased expression of inflammation-related mRNA of TNF-α, IL-6, and IL-1β in the cerebral cortex and hippocampus. Furthermore, LRE ameliorated gut microbiota disorder caused by HFFD by markedly elevating the relative abundances of *Streptococcus* and probiotics such as *Lactococcus*. Concurrently, it reduced the relative abundances of *Helicobacter* and *Clostridium_XIVa.* The levels of tauroursodeoxycholic acid, known for its neuroprotective property, and taurocholic acid, recognized as an anti-inflammatory agent, were significantly enhanced in the hippocampus and cerebral cortex due to the treatment with LRE. In a word, LRE might have the potential to alleviate HFFD-induced cognitive dysfunction by modulating intestinal microbiota and promoting the synthesis of neuroprotective BAs.

## 1. Introduction

Excessive intake of nutritional overload in the form of a high-fat and high-fructose diet (HFFD) was connected with diverse metabolic perturbations, such as hypertension, type 2 diabetes mellitus, cardiovascular disease, obesity, and neuroinflammation [1,2]. Neuroinflammation, recognized as a hallmark of neurodegenerative diseases, particularly Alzheimer’s disease (AD), exerts a significant mental strain on affected individuals due to clinical symptoms such as spatiotemporal disorientation, memory impairment, behavioral disorders, and cognitive deterioration [3]. Extended consumption of HFFD can give rise to metabolic syndrome and cognitive deficits in mice [4]. Moreover, HFFD may trigger robust alterations in the gut microbial community, potentially influencing cognitive functions and behavioral manifestations via the gut–brain axis (GBA), which is strongly associated with neurodevelopment and neurodegeneration [5].

Primary bile acids (BAs) are initially retained in the gallbladder before being released into the small intestine. In the small intestine, the gut microbiome transforms them into secondary BAs [6]. BAs can also act as direct neurotransmitters or neuromodulators, closely linked to the regulation of inflammatory processes in Alzheimer’s disease [7]. The interaction between BAs and gut microbiota relies on a two-way relationship. The gut microbiota dictates BA metabolism, and BAs, in turn, have an impact on the composition of the gut microbiota [8]. The gut microbiota regulates enterohepatic BA metabolism by bio-transforming BAs into products that strongly modulate BA signaling receptors [9]. Notably, key receptors, for instance, Farnesoid X receptor (FXR) and Takeda G-protein receptor 5 (TGR5), along with other nuclear receptors like pregnane X receptor (PXR), liver X receptor (LXR), vitamin D receptor (VDR), and glucocorticoid receptor (GR), are triggered by BAs and showed expression in the brain [6].

The combination of dietary intake and intestinal microbiota interacts with the BA pool, influencing the hydrophobicity, toxicity, and regulation of BAs through biotransformation reactions [9]. The neuroinflammation in mice induced by HFFD could be alleviated using the gut–liver–brain route by regulating BAs and gut microbiota. This involves the increase of tauroursodeoxycholic acid (TUDCA) in the cerebral cortex and hippocampal region, along with alteration in BAs metabolites [1]. During the development of AD, continuous activation of glial cells raises the secretion of pro-inflammatory molecules, exacerbating inflammation and ultimately leading to neuronal degeneration as well as abnormal signaling pathways [10]. Unfortunately, currently available drugs for AD treatment have limited effectiveness, often accompanied by adverse reactions. Hence, there is a growing emphasis on preventing obesity-induced neuroinflammation through dietary control rather than relying solely on medications for AD prevention.

*Lycium ruthenicum* Murray, a unique nutraceutical food used both as fruit and medicine, also known as black wolfberry, is a perennial deciduous shrub that grows in the arid areas of northwest China [11]. The fruits of *L*. *ruthenicum* exhibit a variety of beneficial properties, such as antioxidant, anti-inflammatory, anti-fatigue, and neuroprotection effects [12]. These effects are attributed to its rich nutritional content and functional ingredients, such as anthocyanins, polysaccharides, dietary fiber, organic acids, vitamins, and amino acids [13]. Chen et al. demonstrated that anthocyanins extracted from *L. ruthenicum* could improve memory dysfunction and oxidative stress and mitigate neuroinflammation induced by D-galactose in an adult rat model [14]. Petunidin-3-*O*-[rhamnopyranosyl-(trans-*p*-coumaroyl)]-5-*O*-β-D-glucopyranoside, the major anthocyanin of of *L. ruthenicum*, could relieve cognitive deficits and inhibit neural inflammation in the hippocampus [15]. In recent years, researchers have increasingly focused on the biological activity and the effects of *L. ruthenicum* on gut microbiota [16]. Hereinto, the anthocyanins from *L. ruthenicum* could ameliorate high-fructose diet-induced neuroinflammation by promoting intestinal barrier integrity and enhancing the proliferation of beneficial gut bacteria such as *Lactobacillus* [12]. However, it remains undetermined whether and how the anti-neuroinflammatory activity of the aqueous extract of *L. ruthenicum* (LRE) is related to gut microbiota and BA metabolism. Thus, the current study was designed to explore the neuroinflammatory effect of LRE in HFFD-fed C57BL/6J mice, as well as its association with cognitive impairment and gut microbiome. Additionally, the triple quadrupole liquid ultra-performance liquid chromatography-mass spectrometer (UPLC-MS/MS) was employed to study BA metabolomics profiling.

## 2. Materials and Methods

### 2.1. Preparation of LRE

The fruits of *L. ruthenicum* were supplied by the National Wolfberry Engineering Research Center located in Yinchuan, China. LRE was prepared following our previously reported method with some modifications [17]. In brief, the fruits of *L. ruthenicum* (200 g) were ground and then immersed in boiled distilled water (200 mL), stirring for 3 h. The supernatant was collected, and the residue was subjected to extract twice with boiling water as above. Finally, the three resulting extracts were combined, concentrated with a vacuum concentrator, and lyophilized to afford LRE. The carbohydrate content in LRE was quantified using the phenol-sulfuric acid method [18], with results expressed in glucose equivalents (mg glucose per g LRE). Specifically, crude polysaccharides were extracted twice with 80% ethanol, followed by two washes with water, and then measured with the phenol-sulfuric acid method using glucose as the standard. Additionally, sucrose, glucose, and fructose, the primary water-soluble sugars, were assayed by using high-performance liquid chromatography (HPLC) with an evaporative light scattering detector (ELSD) (Shimadzu, Kyoto, Japan). The HPLC conditions included an Asahipak NH2P-50 4E column (4.6 × 250 mm, 5 µm, Shodex Co., Ltd., Kyoto, Japan), 75% acetonitrile as the mobile phase, a flow rate of 1.0 mL/min, and an oven temperature of 30 °C. For determination of protein content, a BCA assay kit (Nanjing Vazyme Biotech Co., Ltd., Nanjing, China) was utilized. The total phenolic content was assessed using the Folin-Ciocalteu colorimetric method with gallic acid as the standard, while the total flavonoid content was calculated with rutin as the standard and reported in rutin equivalents (mg rutin per g LRE) [19,20].

### 2.2. Animal Experiments

**Figure 1:**
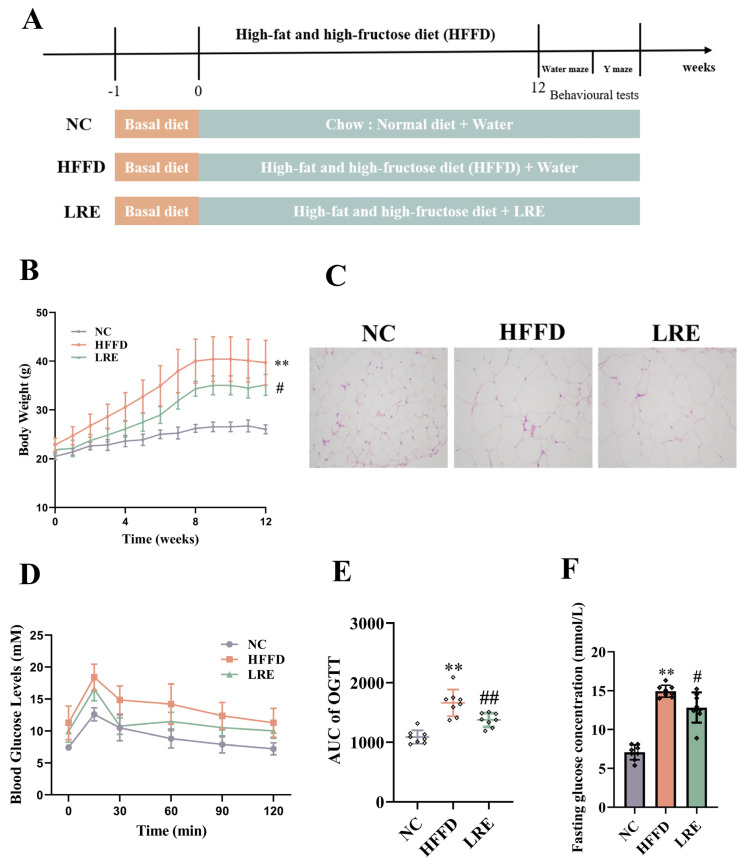
LRE relieved the symptoms of HFFD−induced body weight gain and glucose homeostasis. (**A**) Design of animal experiment protocol. (**B**) Body weight. (**C**) Adipocyte size (epididymal white adipose tissue). (**D**) OGTT. (**E**) AUC of OGTT analysis. (**F**) Fasting glucose concentration. Data are presented as mean ± SEM, *n* = 8. ** *p* < 0.01 compared to the NC group, # *p* < 0.05, ## *p* < 0.01 compared to the HFFD group.

A total of thirty-nine male C57BL/6J mice (6 weeks of age), supplied by Shanghai Slack Laboratory Animal Co., Ltd. (Shanghai, China), were used for animal experiments. Before the experiment, the mice were kept under a specific pathogen-free (SPF) environment with a 12 h daylight cycle. They were allowed free access to water and food for one week at the Animal Center of Nanjing Agricultural University (SYXK<Jiangsu>2021-0086). All the protocols for animal experiments in this study were endorsed by the Ethical Committee of Experimental Animal Center at Nanjing Agricultural University, China (Approval no: NJAU. No 20220221012). The mice were randomly assigned to three groups, with thirteen mice in each group: the normal control (NC) group, the HFFD group, and the LRE group. The mice in the NC group were given the D12450 diet, those in the HFFD group received the D12492 diet, and those in the LRE group were administered HFFD along with 200 mg/kg body weight per day of LRE by gavage for a period of 14 weeks (Figure 1A).

### 2.3. Oral Glucose Tolerance Test (OGTT)

For OGTT, an oral glucose gavage of 2 g/kg was administered after an overnight fasting period of 12 h in the 10th week. Blood samples were taken at 0 (fasting glucose), 15, 30, 60, 90, and 120 min, and measured immediately using a glucometer. The total area under the curve (AUC) of OGTT was calculated by using the trapezoid method [21].

### 2.4. Testing of Serum Samples

The concentrations of tumor necrosis factor-alpha (TNF-α), lipopolysaccharide (LPS), and interleukin (IL)-6 in serum were determined by utilizing commercially available ELISA kits obtained from the Institute of Nanjing Jiancheng Bioengineering (Nanjing, China).

### 2.5. Behavior Assessment

#### 2.5.1. Morris Water Maze (MWM) Assessment

The MWM test was used to evaluate the spatial cognition of mice as described in previous study [22]. During the four consecutive days of training trials, the mice were randomly assigned to one of the four quadrants of the pool for each trial to avoid any bias in the starting position. Thereafter, the time spent by each mouse in locating the platform was documented, with a maximum trial duration of 90 s. In case the mouse was unable to reach the concealed platform during 90 s, it was guided to the platform and allowed to remain on it for 15 s. The platform was removed on the fifth day of the study for spatial probe experiment, enabling each mouse to locate the platform within 90 s. The following indicators of MWM performance, including the number and distance crossings of the platform and the amount of time spent in the target area of the platform, were recorded. Experimenters were blinded to the treatment groups during the assessment to minimize potential biases.

#### 2.5.2. Y-Maze Assessment

The Y-maze is a three-arm horizontal maze designed to evaluate the performance of spatial learning and memory abilities of rodents through calculating spontaneous alteration behavior. Typically, normal rodents preferred to experience an arm of the maze other than the one they entered on their previous attempt. The Y-maze test in our study was performed in accordance with previous descriptions [22], and a randomization method was used to assign the starting arm for each mouse to avoid bias. Additionally, the experimenters were blinded to the treatment groups to ensure unbiased results.

### 2.6. Analysis of RT-qPCR

The RT-qPCR analysis was undertaken following the reported method [16]. Approximately 20 mg of liver, cerebral cortex, or hippocampus tissue was weighed and homogenized in a lysis buffer. The total RNA was then extracted using a commercial RNA extraction kit (Nanjing Vazyme Biotech Co., Ltd.) according to the manufacturer’s instructions, which included steps for lysis, binding, washing, and eluting in RNase-free water. RNA concentration and quality were assessed using a NanoDrop 2000 spectrophotometer (Thermo Scientific, Waltham, MA, USA). Total RNA was transcribed to cDNA in reverse using a reverse transcription kit (HiScrip III RT SuperMix) purchased from Nanjing Vazyme Biotech Co., Ltd. RT-qPCR was performed using a ChamQ Universal SYBR qPCR Master Mix kit (Nanjing Vazyme Biotech Co., Ltd.) on Quant Studio 5 (ABI, Los Angeles, CA, USA). The primer sequences are shown in Table 1.

### 2.7. Histological Analysis and Immunofluorescence Analysis

The livers and tissues of the brain, adipose, and intestines from mice were treated with 4% paraformaldehyde for fixation and then paraffin-embedded and processed into slices with a thickness of 4 µm. The slices were stained using hematoxylin and eosin (H&E) for morphological observations. Images were captured by a Nikon microscope (Tokyo, Japan) and an inverted fluorescence microscope (Tokyo, Japan).

### 2.8. Analysis of BAs

BAs standards, cholic acid (CA), deoxycholic acid (DCA), tauro-α-muricholic acid, α-muricholic acid (αMCA), β-muricholic acid (βMCA), ω-muricholic acid (ωMCA), chenodeoxycholic acid (CDCA), hyodeoxycholic acid (HDCA), ursodeoxycholic acid (UDCA), lithocholic acid (LCA), taurochenodeoxycholic acid (TCDCA), taurocholic acid (TCA), TUDCA, glycodeoxycholic acid (GDCA), and glycocholic acid (GCA) were procured from Beijing Solarbio Science & Technology Co., Ltd. (Beijing, China). Additionally, tauro-α-muricholic acid (TαMCA), tauro-β-muricholic acid (TβMCA), taurodeoxycholic acid (TDCA), glycochenodeoxycholic acid (GCDCA), CA-d_4_, DCA-d_4_, and GCDCA-d_4_ were purchased from Shanghai ZZBIO Co., Ltd. (Shanghai, China). The cortex, hippocampus and liver were collected and immediately frozen in dry ice, and the samples were stored at 80 °C until analysis. BAs in serum, liver, and brain were measured according to previously reported methods with slight modifications [23,24]. Briefly, an aliquot of 100 µL of plasma was mixed with 200 µL acetonitrile (LC-MS grade), which contained internal standard (CA-d_4_, GCDCA-d_4_ and DCA-d_4_). Intestinal content, cortex, hippocampus, and liver samples were homogenized and extracted in acetonitrile containing deuterated internal standards (CA-d_4_, GCDCA-d_4_ and DCA-d_4_). Then, the supernatant was collected by centrifugation at 12,000 rpm and 4 °C for 20 min. The solvent of the supernatant was evaporated by termovap sample concentrator. The dried powder was reconstituted with 100 µL pure acetonitrile and filtered through a 0.45 µm membrane. BAs were then analyzed by 5500 triple quadrapole (AB SCIEX, Foster City, CA, USA). Each individual BA was identified based on the retention time of the corresponding standard and quantified according to the ratio of the peak area of the internal standard to that of the target compound.

### 2.9. Gut Microbiota Analysis

The analysis of the microbial community in mice feces through 16S rRNA was carried out as detailed in our earlier study [1]. Genomic DNA from feces was extracted using the QIAamp Fast DNA Stool Mini Kit (Qiagen, Venlo, The Netherlands). The obtained DNA was amplified by PCR targeting the selected V3–V4 variable regions with the primer sequence of 341F (5′-CCTACGGGNGGCWGCAG-3’) and 805R (5′-GACTACHVGGGTATCTAATCC-3’). For each fecal sample, high-throughput sequencing analysis was conducted on the Illumina NovaSeq 6000 sequencer platform from Illumina (San Diego, CA, USA).

### 2.10. Statistical Analysis

All data are presented as mean ± standard deviation (SD). In addition to the data on BAs, other data were determined through Student’s test. For BAs, each of the metabolites was compared by using a Mann–Whitney *U* test. Statistical significance between means was determined by one-way ANOVA by using GraphPad Prism 6.0. Differences were considered statistically significant when *p* < 0.05.

## 3. Results

The prepared LRE mainly consisted of 578.9 ± 4.11 mg/g water-soluble carbohydrates, 203.70 ± 26.42 mg/g polysaccharides, 54.28 ± 0.22 mg/g polyphenols, 91.60 ± 10.35 mg/g crude proteins, and 1.48 ± 0.13 mg/g flavonoids. In addition, LRE consisted of fructose, glucose, and sucrose at concentrations of 81.17 ± 0.32, 184.86 ± 3.39, and 76.81 ± 0.92 mg/g, respectively.

### 3.1. LRE Reduced Weight Gain in HFFD-Fed Mice

The body weight trends in mice were monitored every week to assess the impact of LRE on the weight of HFFD-fed mice (Figure 1B). In comparison with the NC group, mice fed with HFFD had a remarkable rise in body weight (*p* < 0.01). Significantly, LRE administration notably decreased the weight gain of mice (*p* < 0.05) compared with the HFFD group. The HFFD group showed an enlargement of adipocyte size in the epididymal adipose tissue, which was effectively reduced by the LRE administration (Figure 1C). OGTT was utilized to investigate the hypoglycemic effect of LRE on HFFD-fed mice. The blood glucose levels of mice in the HFFD group were higher than those in the NC group at 15, 30, 60, 90, and 120 min. The LRE treatment caused a notable decrease in AUC of OGTT values compared with the HFFD group (Figure 1D,E). In addition, the LRE administration significantly lowered the fasting blood glucose level in HFFD-fed mice (Figure 1F, *p* < 0.05).

### 3.2. Effects of LRE on Preventing HFFD-Induced Cognitive Deficits

**Figure 2:**
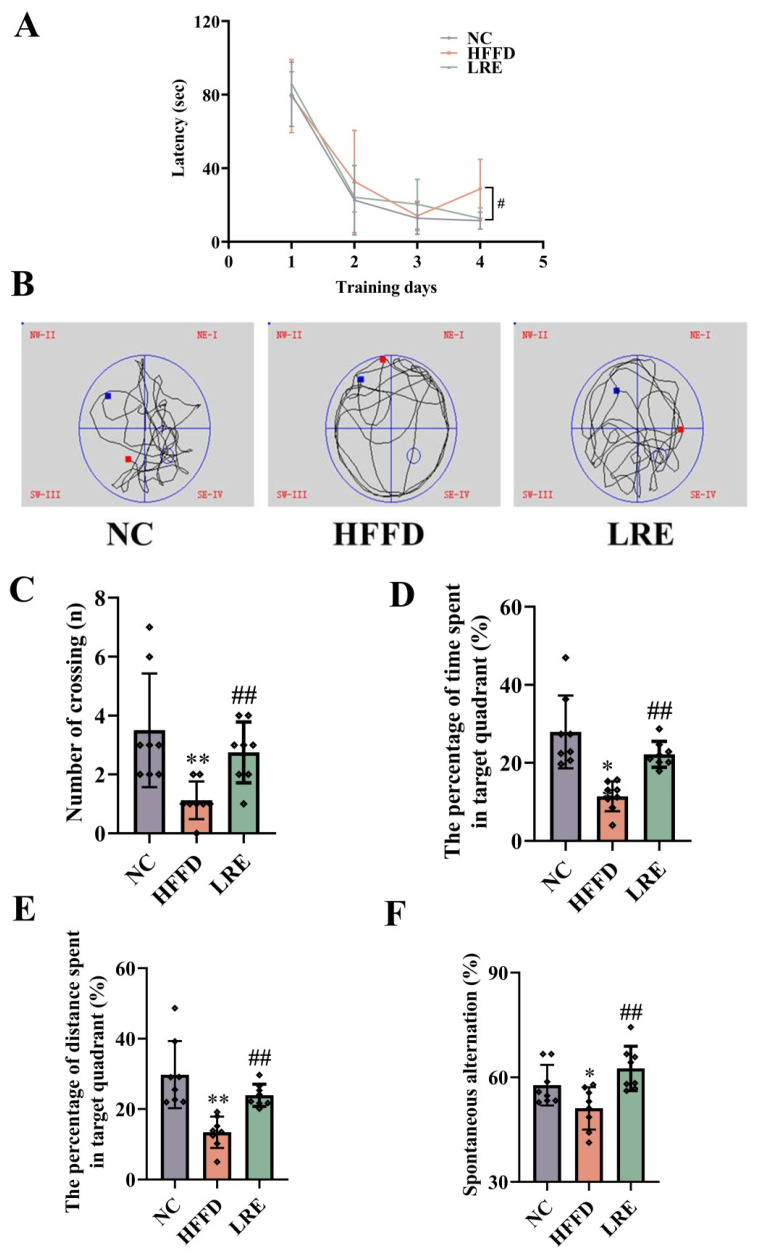
LRE alleviated HFFD-induced cognitive dysfunction. (**A**) Time of the latency to the platform. (**B**) Representative swimming paths (probe trial). (**C**) The number of platform crossings. (**D**) The percentage of time spent in the target zone. (**E**) The percentage of distance spent in the target quadrant. (**F**) Spontaneous alternation (Y maze). * *p* < 0.05, ** *p* < 0.01 compared to the NC group, # *p* < 0.05, ## *p* < 0.01 compared to the HFFD group. The blue point is the starting point of the MWM experiment, and the red point is the end point.

The MWM experiment was performed to evaluate the influences of LRE on spatial learning, memory, and cognition via measuring trajectory diagram, escape latency, crossing numbers, the proportion of residence distance, and duration spent in the target quadrant (Figure 2). The LRE group showed a remarkable reduction in escape latency on day 4 compared with the HFFD group (Figure 2A, *p* < 0.05). After the usual test, a probe trial was employed to evaluate how well the mice remembered the platform’s location (Figure 2B). The mice treated by HFFD, with fewer number of platform crossings, had a shorter duration in the target area (11.44%, *p* < 0.01, Figure 2C,D) in contrast to the NC group. As depicted in Figure 2E, the LRE administration significantly reversed the distance of the HFFD group spent swimming in the target zone (13.43% (*p* < 0.01) to 23.93% (*p* < 0.01)). The behavior of spontaneous alternation exhibited in the Y-maze was affected by LRE. The percentage of spontaneous alternation in the HFFD group was notably lower than that in the NC group, and the LRE administration markedly improved this reduction in spontaneous alternation (*p* < 0.01, Figure 2F). These findings revealed that the learning and memory capabilities of the LRE group and the NC group were superior to those of the HFFD group.

### 3.3. LRE Attenuated Microglia Activation and Neuroinflammation Induced by HFFD

**Figure 3:**
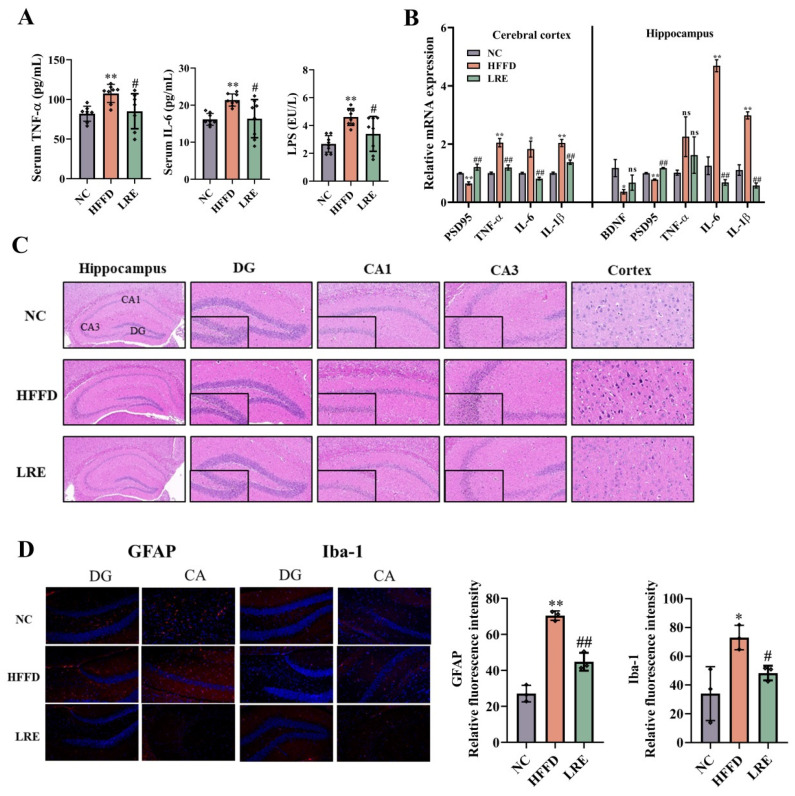
LRE alleviated HFFD-induced inflammation and neural neuroinflammation. (**A**) Contents of TNF-α, IL-6 levels, and LPS in serum (*n* = 8). (**B**) TNF-α, IL-6, IL-1β, and PSD-95 mRNA levels in the cerebral cortex and hippocampus (*n* = 6). (**C**) Representative H&E staining of brain tissue in the CA1, CA3, and DG of the hippocampus and cortex. (**D**) Representative images and quantitative analysis of GFAP, Iba-1 and PSD-95 in hippocampus by immunofluorescence analysis (*n* = 3). Data are presented as mean ± SD, *n* = 8. * *p* < 0.05, ** *p* < 0.01 compared to the NC group, # *p* < 0.05, ## *p* < 0.01 compared to the HFFD group, ns stands for non-significant compared to the HFFD group.

The concentrations of serum LPS, TNF-α, and IL-6 were determined in this study. As shown in Figure 3A, in comparison to the NC group, HFFD brought about a remarkable rise in the contents of TNF-α, IL-6, and LPS (*p* < 0.01). However, this detrimental effect was significantly reversed in the LRE group (*p* < 0.05). Thus, LRE appeared to reduce serum-inflammatory factor levels and improve serum lipid indices. Subsequently, the expression levels of mRNA for pro-inflammatory cytokines (TNF-α, IL-6 and IL-1β) in the brains of mice were measured by PCR. The results indicated that the levels of TNF-α, IL-6, and IL-1β in the cerebral cortex were considerably higher in the HFFD group than those in the NC group. However, the LRE consumption significantly inhibited the elevation of TNF-α, IL-6, and IL-1β. LRE remarkably decreased the expression of IL-6 and IL-1β in the hippocampus when contrasted with the HFFD group (Figure 3B). To investigate the effect of LRE on improving the histopathology of the hippocampus and cortex induced by HFFD in mice, H&E staining was employed to inspect the morphological characteristics of neurons in the cortex as well as in the CA1, CA3, and DG (dentate gyrus) regions of the hippocampus. The neurons were arranged in a disorderly manner (particularly in the CA1 and CA3 regions). Pyknotic hyperchromatic nuclei were observed in the DG region of the HFFD group in comparison with the NC group, while this phenomenon was improved due to the treatment with LRE. Similarly, in the HFFD group, damaged neurons were detected in the cortex. In the LRE group, these neurons displayed a normal distribution (Figure 3C). Immunofluorescence assay was utilized to reveal the expression and location of glial fibrillary acidic protein (GFAP) and ionized calcium-binding adapter molecule 1 (Iba-1) in the hippocampus. As shown in Figure 3D, in contrast to the NC group, HFFD led to a dramatic increase in the expressions of GFAP and Iba-1. The elevated expressions of GFAP and Iba-1 indicated the persistent activation of glial cells, which might release proinflammatory cytokines, aggravating neuroinflammation. However, the LRE administration significantly reduced the immunofluorescence intensity of GFAP (*p* < 0.01) and Iba-1 (*p* < 0.05) in the hippocampus compared with the HFFD group. These findings implied the potential neuroprotective function of LRE on mice induced by HFFD.

### 3.4. Effects of LRE on Hepatic and Feces BAs Levels in HFFD-Fed Mice

**Figure 4:**
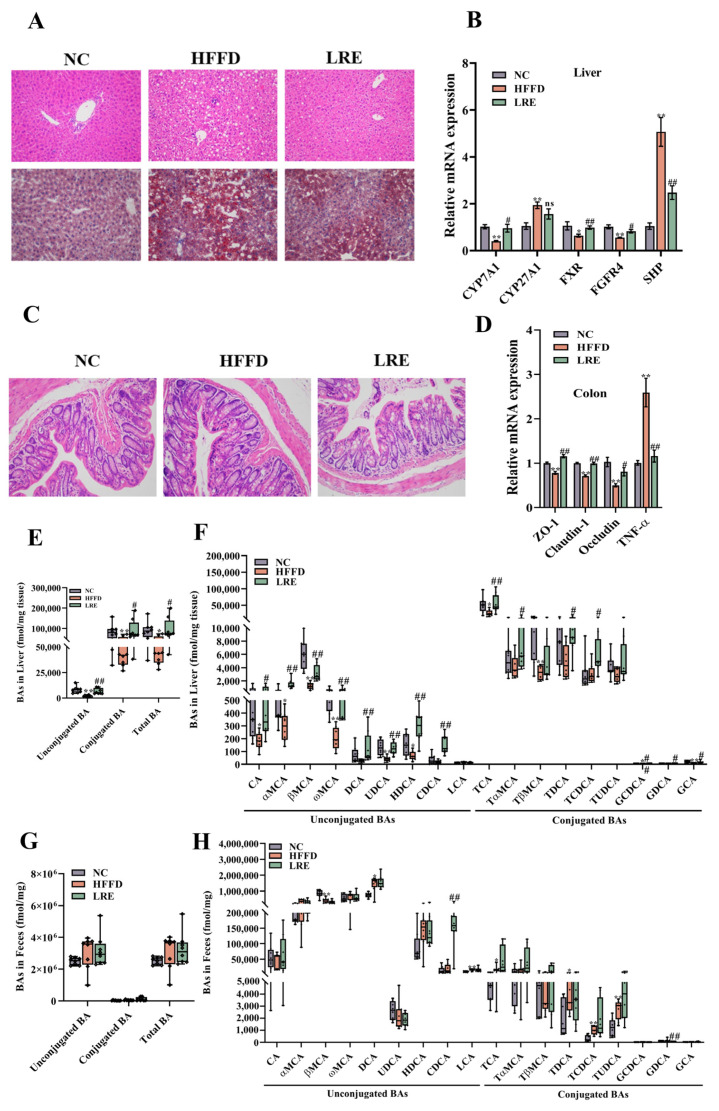
Effects of LRE on levels of BAs in liver and gut. (**A**) Histological sections of liver tissues (*n* = 3). (**B**) CYP7A1, CYP27A1, FXR, FGFR4, and SHP relative mRNA expression levels in the liver. (**C**) Histological sections of colonic tissues stained with H&E (*n* = 3). (**D**) The mRNA levels of ZO-1, claudin-1, occludin, and TNF-α in the colon (*n* = 6). (**E**) Total BAs levels in the liver (*n* = 8). (**F**) The levels of conjugated and unconjugated BAs in the liver (*n* = 8). (**G**) Total BAs levels in the feces (*n* = 8). (**H**) The levels of conjugated and unconjugated BAs in the feces (*n* = 8). Data are presented as mean ± SD, *n* = 8. * *p* < 0.05, ** *p* < 0.01 compared to the NC group, # *p* < 0.05, ## *p* < 0.01 compared to the HFFD group.

The results of liver tissue stained with H&E and Oil Red O indicated that LRE mitigated HFFD-induced liver fat accumulation and inflammation (Figure 4A). In contrast to the HFFD group, the administration of LRE markedly enhanced the mRNA levels of expression of cholesterol 7α-hydroxylase (CYP7A1), FXR, and FGFR4. Meanwhile, the LRE intervention notably suppressed the level of SHP expression. Intriguingly, the rising expression of CYP27A1 induced by HFFD was downregulated by the LRE intervention, but the difference was not significant (Figure 4B). Microscopic examination of H&E-stained histological sections of colonic tissues is shown in Figure 4C. The NC group showed intact mucosal epithelial cells with no observable colonic lesions. In contrast, colonic inflammatory changes and tissue impairment in the mice in the HFFD group were observed, but this status could be attenuated by LRE administration. The expression levels of ZO-1, claudin-1, and occludin mRNA were significantly upregulated in the LRE group as compared to the HFFD group (Figure 4D). The mRNA level of TNF-α was markedly higher in the HFFD-fed mice in comparison with the NC group (*p* < 0.01) but after LRE treatment it was notably downregulated when compared to the HFFD group (*p* < 0.01). To investigate the influence of HFFD feeding on enterohepatic circulation of BAs, the types and concentrations of BAs in the liver and feces in the NC, HFFD, and LRE groups were analyzed. As shown in Figure 4E, the total concentration of BAs level in the liver, comprised of unconjugated BAs and conjugated BAs, in the HFFD group was strikingly less than that of the NC group (*p* < 0.05). Moreover, in the LRE group, the total BAs level was markedly superior to that of the HFFD group (*p* < 0.05). Compared with the NC group, the treatment of LRE profoundly increased the concentrations of conjugated BAs (TCA, TαMCA, TDCA, and TCDCA) as well as unconjugated BAs such as CA, αMCA, βMCA, ωMCA, DCA, UDCA, HDCA, and CDCA in the liver of mice (Figure 4F). In this study, taurine-conjugated BAs were the predominant BAs in the liver, and among these levels, TCA was the highest. The levels of taurine-conjugated BAs (TCA, TαMCA, TCDCA, and TUDCA) in the feces were increased in the LRE group compared with the HFFD group. Nevertheless, the difference did not show statistical significance. The abundances of GDCA and CDCA tended to be elevated significantly in the LRE group in contrast to the HFFD group (Figure 4H).

### 3.5. Effects of LRE on Serum and Brain BAs Levels in HFFD-Induced Mice

**Figure 5:**
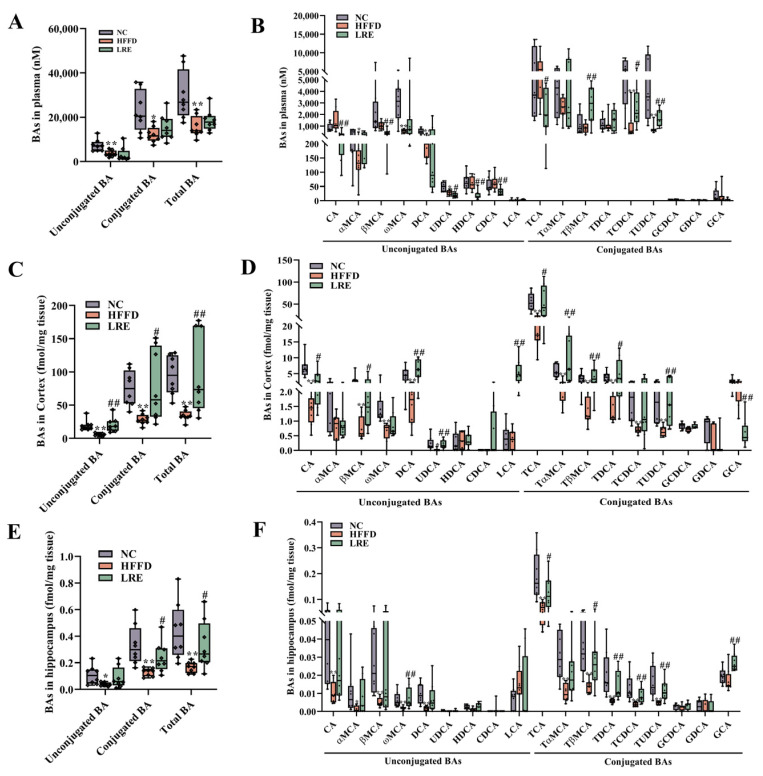
LRE-altered BAs profile in plasma and brain. (**A**) Total plasma BA levels (*n* = 8). (**B**) The levels of conjugated and unconjugated BAs in the plasma (*n* = 8). (**C**) Total cortex BA levels (*n* = 8). (**D**) The levels of conjugated and unconjugated BAs in the cortex (*n* = 8). (**E**) Total hippocampus BA levels (*n* = 8). (**F**) The levels of conjugated and unconjugated BAs in the hippocampus (*n* = 8). Data are presented as mean ± SD, *n* = 8. * *p* < 0.05, ** *p* < 0.01 compared to the NC group, # *p* < 0.05, ## *p* < 0.01 compared to the HFFD group.

The results revealed that HFFD induced a significant decline in the total BAs level in the serum compared with the NC group (Figure 5A). A close inspection of changes of individual BA in serum showed that the LRE treatment decreased the contents of CA, βMCA, UDCA, CDCA, HDCA, and TCA. In addition, the LRE treatment significantly increased serum tauro-BAs levels, which were represented by TβMCA, TCDCA, and TUDCA levels (Figure 5B). In comparison with the HFFD group, the LRE group considerably enhanced the total BAs in the cortex (Figure 5C). Specifically, the LRE treatment significantly upregulated cortex concentrations of conjugated BAs (TCA, TαMCA, TβMCA, TDCA, and TUDCA) and the concentrations of unconjugated BAs (CA, βMCA, DCA, UDCA, and LCA) in contrast to the HFFD group (Figure 5D). The total levels of BAs in the hippocampus were improved in the LRE group (Figure 5E). However, there was a marked decrease in the levels of conjugated BAs in the hippocampus, namely TCA, TαMCA, TβMCA, TDCA, TCDCA, and TUDCA, which was related to HFFD intervention in comparison with the NC group. Meanwhile, a significant decrease in unconjugated BAs (CA, αMCA, βMCA, ωMCA, and DCA) level was noted in the HFFD group (Figure 5F). In addition, the LRE group showed dramatic increases in the levels of ωMCA, TCA, TβMCA, TDCA, TCDCA, TUDCA, and GCA compared with the HFFD group. Our findings suggested that LRE intervention had the potential to attenuate HFFD-induced disorder of BA metabolism.

### 3.6. Effects of LRE on Gut Microbiota in HFFD-Induced Mice

**Figure 6:**
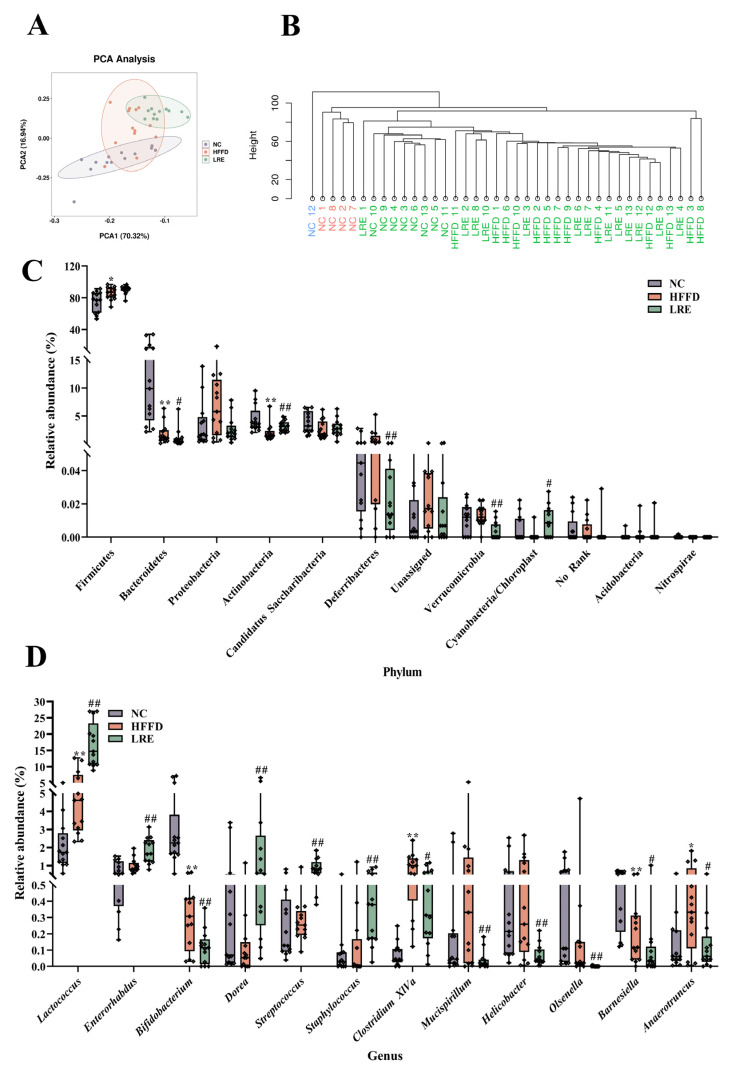
LRE ameliorated gut microbiota dysbiosis induced by the HFFD. (**A**) PCA of gut microbiota (*n* = 10). (**B**) Hierarchical cluster analysis (HCA) at the OTU level in the different groups (*n* = 13). (**C**) Relative abundance of gut microbial composition at phylum level. (**D**) Relative abundance of gut microbial composition at genus level. Data are presented as mean ± SD, *n* = 8. * *p* < 0.05, ** *p* < 0.01 compared to the NC group, # *p* < 0.05, ## *p* < 0.01 compared to the HFFD group.

**Figure 7:**
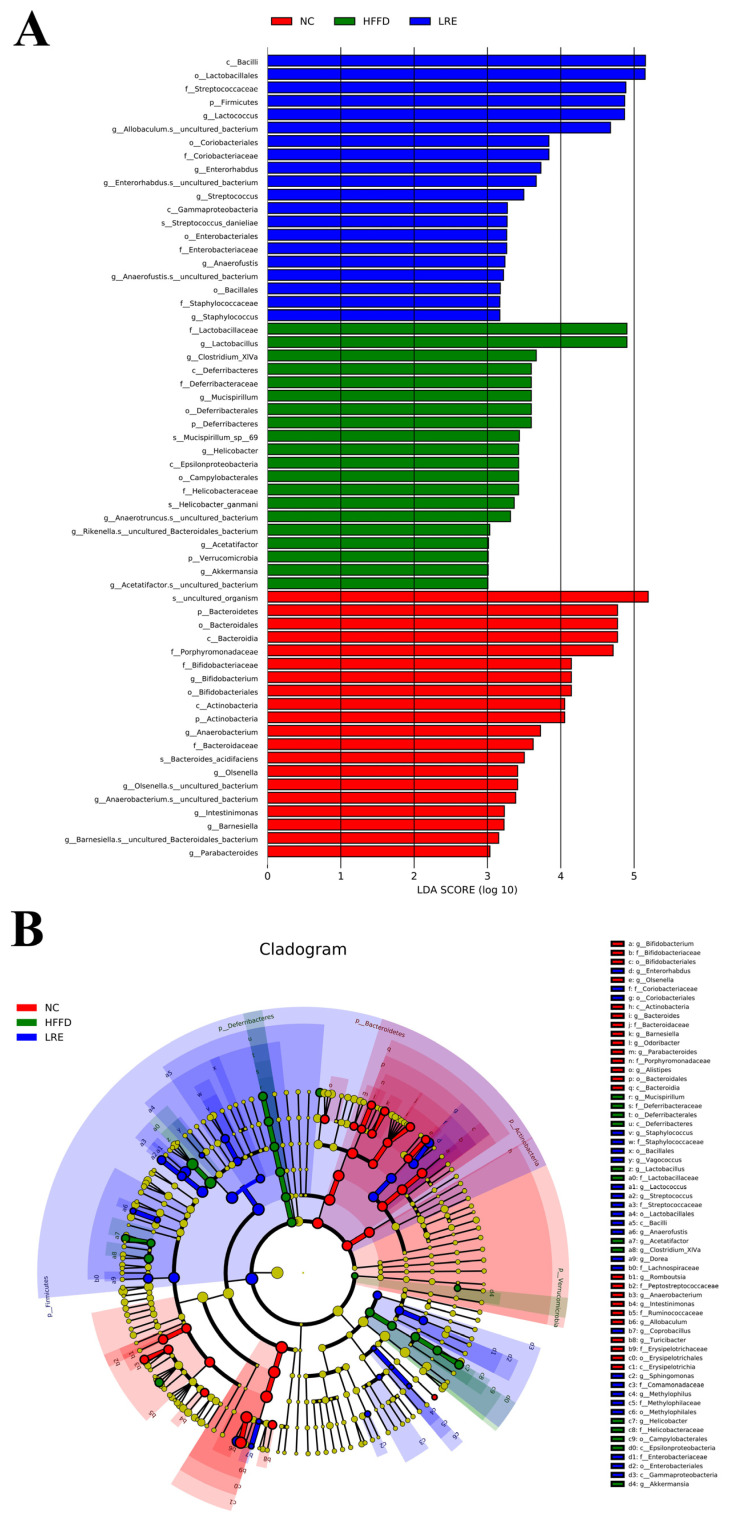
Effects of LRE on gut microbiota in HFFD-induced mice. (**A**) Enriched taxa of three groups (LDA > 3). (**B**) LEfSe taxonomic cladogram (LDA > 3).

The impact of LRE on the gut microbiota composition was studied through sequencing the bacterial 16S rRNA in feces. The principal component analysis (PCA) and hierarchical cluster analysis (HCA) were employed to perform the relative distribution profiles in β-diversity. The percentage variation of the PC1 and PC2 components accounted for 70.32% and 16.94% (Figure 6A), respectively. As expected, the microbial community changed greatly in the HFFD group in comparison with the NC group, but the LRE intervention ameliorated the dysbiosis induced by HFFD. Hierarchical clustering at the OTU level is in accordance with the results of PCA, suggesting that LRE treatment could regulate the community of gut microbiota (Figure 6B). The microbial structure at phylum and genus levels was illustrated. In terms of level, the intestinal microbes of the three groups of mice primarily consisted of Firmicutes, Bacteroidetes, Proteobacteria, Actinobacteria, and Candidatus_Saccharibacteres. Actinobacteria and Cyanobacteria/Chloroplast were increased significantly, while Bacteroidetes, Deferribacteres, and Verrucomicrobia declined markedly in the LRE group when compared to the HFFD group (Figure 6C). Besides, at the genus level, an upward trend in *Lactococcus, Enterorhabdus*, *Bifidobacterium*, *Dorea*, *Streptococcus*, and *Staphylococus* was presented in the LRE group compared with the HFFD group (*p* < 0.01). In comparison with the HFFD group, the relative abundances of *Clostridium_XlVa*, *Mucispirillum*, *Helicobacter*, *Olsenella*, *Barnesiella*, and *Anaerotruncus* were remarkably decreased in the LRE group (Figure 6D). The microbial variance among the NC, HFFD, and LRE groups was further compared using linear discriminant analysis (LDA) effect size (LEfSe) (LDA > 3, Figure 7A). It was found that *Lactococcus, Enterorhabdus, Streptococcus, Anaerofustis,* and *Staphylococcus* were enriched in the LRE group. It could be obviously found that *Lactobacillus, Clostridium_XIVa, Mucispirillum, Helicobacter, Acetatifactor,* and *Akkermansia* were enriched in the HFFD group. The findings revealed that *Bifidobactertium, Anaerobacterium, Glsenella, Intestinimonas, Barnesiella,* and *Parabacteroides* were enriched in the NC group. The LRE intervention could significantly adjust the gut microbiota.

## 4. Discussion

Accumulating evidence has revealed that consuming a HFFD can cause neuroinflammation in the brain of mice, which in turn impairs their learning and memory functions [22]. In this research, the neuroprotective effects of LRE on HFFD-induced neuroinflammation were investigated. HFFD treatment resulted in weight gain in mice, a significant increase in blood glucose, impairment of working memory and spatial memory, as well as inflammation in the hippocampus and cortex. HFFD also triggered changes in BA metabolism and caused dysbiosis in the gut microbiota of mice. *L. ruthenicum*, a well-known Chinese herbal medicine with abundant anthocyanins, has been proven to have potential health-enhancing effects [12]. Nevertheless, a limited number of studies have centered on the actual bioactivities of *L. ruthenicum* as an edible whole berry. Moreover, it remains unclear whether *L. ruthenicum* regulates HFFD-induced neuroinflammation by BA metabolism and gut microorganism. In the current study, we conducted an exploration of the anti-neuroinflammatory potency of LRE. As expected, LRE played a crucial role in preventing HFFD-induced cognitive decline.

There is growing evidence showing that obesity has adverse effects on cognitive function [1,12]. In the behavioral experiment, HFFD impaired the cognitive function of mice, and LRE intervention alleviated the cognitive dysfunction caused by HFFD. H&E staining showed extensive neuronal damage in the HFFD group, which was effectively reversed by LRE. Tozuka et al. [25] reported that mice with cognitive deficits exhibited downregulation of BDNF and PSD95 in the cortex and hippocampus in the group of the model. Consistent with these findings, our study revealed that LRE intervention could increase the level of neurotrophic factor BDNF and PSD95 in the brain of mice. Studies have demonstrated that learning ability is influenced by the activity of BDNF. As a crucial neurotrophin, BDNF is a major factor in the consequences of cognitive plasticity [26].

Cognitive impairment in AD and decline in cognitive ability are related to alterations in the gut microbiome [27]. Diet-induced obesity in mice following a HFFD was linked to an elevation in Firmicutes and a lower abundance of Bacteroidetes, which is consistent with our current research. In our study, LRE altered the gut microbiota of mice. 16S rRNA gene sequencing analysis revealed that in the LRE group, the relative abundances of *Lactococcus, Enterorhabdus, Dorea, Streptococcus,* and *Staphylococus* increased significantly, while *Bifidobacterium, Clostridium_XlVa*, *Mucispirillum*, *Helicobacter, Olsenella, Barnesiella*, and *Anaerotruncus* decreased significantly. Yang et al. [28] demonstrated that inulin could recover gut microbiota balance to attenuate alcoholic liver disease by increasing the abundance of beneficial bacteria *Lactococcus* at the genus level. In addition, Su et al. [29] discovered that the relative abundance of *Lactococcus* increased after supplementation with quercetin in obese mice. The engineered strain MG136-pMG36e-GLP-1 was assessed as having a neuroprotective effect on AD and Parkinson’s disease mice due to the probiotic properties of *Lactococcus lactis* MG1363 and the continuously generated glucagon-like peptide-1 (GLP-1) from the engineered strain [30]. Wang and colleagues announced that the abundance of *Clostridium_XlVa* in healthy individuals was reduced at the genus level compared with obstructive sleep apnea patients [31]. A study reported that *Clostridium_XlVa* was linked to irritable bowel syndrome (IBS). Compared with the control, the microbiota of IBS patients showed a 1.5-fold rise in the amount of *Clostridium_XlVa* [32]. Liu and colleagues stated that *Helicobacter* infection could cause an elevated rate of incident diabetes. In our study, the relative abundances of *Clostridium_XlVa* and *Helicobacter* in the mice were substantially reduced due to the treatment with LRE in comparison with HFFD [33].

Present in the brain is 20% of the body’s cholesterol, and BA generation is a crucial mechanism for removing cholesterol [34]. As the rate-limited enzyme in the classical pathway, CYP7A1 converts cholesterol into BAs and plays a crucial role in the regulation of cholesterol, glucose metabolism, and BAs homeostasis [35]. Li et al. demonstrated that the induction of hepatic CYP7A1 had the potential to prevent obesity, fatty liver, and insulin resistance induced by a high-fat diet, thereby presenting a promising therapeutic alternative for metabolic disorders in humans [36]. In this study, LRE significantly upregulated the relative mRNA expression of hepatic CYP7A1. A recent investigation identified that there are 20 diverse types of BAs in the CNS [6]. TUDCA, a hydrophilic BA produced in the liver, has shown therapeutic potential for preventing and treating AD in mice [37]. Elia et al. proposed that TUDCA might possess promising neuroprotective activity in patients with a neurodegenerative disease [38]. Wu et al. demonstrated that TUDCA improved LPS-induced cognitive dysfunction and neuroinflammation associated with the TGR5-mediated NF-κB signaling pathway [39]. Additionally, Dionisio et al. assessed the protective impacts of TUDCA in APP/PS1 mice, observing a decrease in glial activation as well as lowered expression of proinflammatory cytokine messenger RNA [10]. As expected, the levels of TUDCA in the cortex and hippocampus reduced notably in the HFFD-treated mice, which were reversed in the LRE group. TCA served as an efficient natural anti-inflammatory substance, effectively inhibiting the cellular secretion of pro-inflammatory cytokines in LPS-induced zebrafish and macrophages [40]. In our study, the LRE intervention notably enhanced TCA concentrations in the feces, cortex as well as the hippocampus. The BAs with higher content in the liver, plasma, cortex, and hippocampus were primarily TCA. A rise in CA levels in the cortex and hippocampus was observed in the LRE group. CA was shown to strongly promote the expression of growth factors in the brain of mice with cognitive deficits triggered by ibotenic acid, suggesting its significance in searching for effective drugs for AD [41]. Moreover, circulating CDCA in serum could enhance the permeability of the blood–brain barrier during obstructive cholestasis through the impairment of tight junction, potentially inducing neurotoxicity [42]. In our experiment, LRE significantly decreased circulating CDCA levels in serum. Administering DCA to HFD-fed foz/foz mice could increase TGR5 and FXR signaling, improving the metabolic disorder and preventing steatosis [43]. LRE dramatically increased the level of DCA in comparison with the HFFD group in the liver and cortex (*p* < 0.01). Our study revealed an elevated total hepatic BAs level, as well as increased total cortex and hippocampus BAs, indicating enhanced BAs synthesis due to the LRE treatment. BAs seemed to perform a crucial function in modulating the gut microbiota. A minor cluster of intestinal species of the genus *Clostridium*, such as *Clostridium group XVIa*, has been reported to be able to produce secondary BAs [44]. CA consumption led to phylum-level changes in the gut microbiome, with Firmicutes greatly enlarging [45]. A previous study suggested that *Lactobacillus* and *Bifidobacterium* in the human gut were controlled to some extent by the concentration of CA [46]. Our present results indicated that LRE supplementation raised the relative abundances of *Lactobacillus* and decreased the relative abundances of *Clostridium_XlVa*. Therefore, the regulation of BA metabolism by LRE might be achieved by enriching intestinal *Lactobacillus* and reducing the relative abundances of *Clostridium_XlVa* to alleviate neuroinflammation in HFFD-induced mice. In addition, the metabolism of BAs in the gut was realized through the synergistic action of multiple bacteria.

## 5. Conclusions

In summary, our findings showed that LRE could improve neuroinflammatory injury and memory impairments caused by HFFD in mice. Meanwhile, LRE attenuated the concentrations of inflammatory cytokines (TNF-α, IL-6, and IL-1β) in the cortex and hippocampus. Simultaneously, it enhanced the BDNF expression in the hippocampus. LRE modulated the dysbiosis of gut microbiota by restoring the relative abundances of *Lactobacillus* and downregulating *Clostridium_XlVa.* Importantly, our results demonstrated that LRE significantly increased the concentrations of TCA and TUDCA in the hippocampus and cerebral cortex, thereby exerting anti-inflammatory and anti-neuroinflammatory impacts, respectively. Furthermore, the administration of LRE increased the total BAs in the liver in comparison with the HFFD group. Our findings suggested the potential application of LRE as an active substance for the prevention of neuroinflammation induced by HFFD.

## Figures and Tables

**Table 1:** Primer sequences utilized in the RT-qPCR experiments.

Target Gene	Primer	Sequence (5′-3′)
TNF-α	forward	CTCATGCACCACCATCAAGG
	reverse	ACCTGACCACTCTCCCTTTG
IL-1β	forward	AGCTTCAAATCTCGCAGCAG
	reverse	TCTCCACAGCCACAATGAGT
IL-6	forward	CTCTGGCGGAGCTATTGAGA
	reverse	AAGTCTCCTGCGTGGAGAAA
PSD-95	forward	TCTGTGCGAGAGGTAGCAGA
	reverse	AAGCACTCCGTGAACTCCTG
CYP7A1	forward	AGCAACTAAACAACCTGCCAGTACTA
	reverse	GTCCGGATATTCAAGGATGCA
CYP27A1	forward	GCCTCACCTATGGGATCTTCA
	reverse	TCAAAGCCTGACGCAGATG
FXR	forward	AGGAGCCCCTGCTTGATGT
	reverse	GCGGGTTCTCAGGCTGGTA
FGFR4	forward	GGCTCCATGACCGTCGTACA
	forward	ATGACCACTCGAGGAGCTGC
SHP	reverse	AGGGTAGAGGCCATGAGGAG
	forward	ACGATCCTCTTCAACCCAGA
ZO-1	reverse	TGAGTGCGTTTCTCTCCCTT
	forward	CCCTCTGTGTTCCTCATGGT
Occludin	forward	AGCACTTAACCTGCCTGGAT
	reverse	AGCCTGTGGAAGCAAGAGAT
Claudin-1	forward	AGCTGCCTGTTCCATGTACT
	forward	CTCCCATTTGTCTGCTGCTC
GAPDH	reverse	GGACTTACAGAGGTCCGCTT
	forward	CTATAGGGCCTGGGTCAGTG

## Data Availability

The original contributions presented in the study are included in the article, further inquiries can be directed to the corresponding authors.
